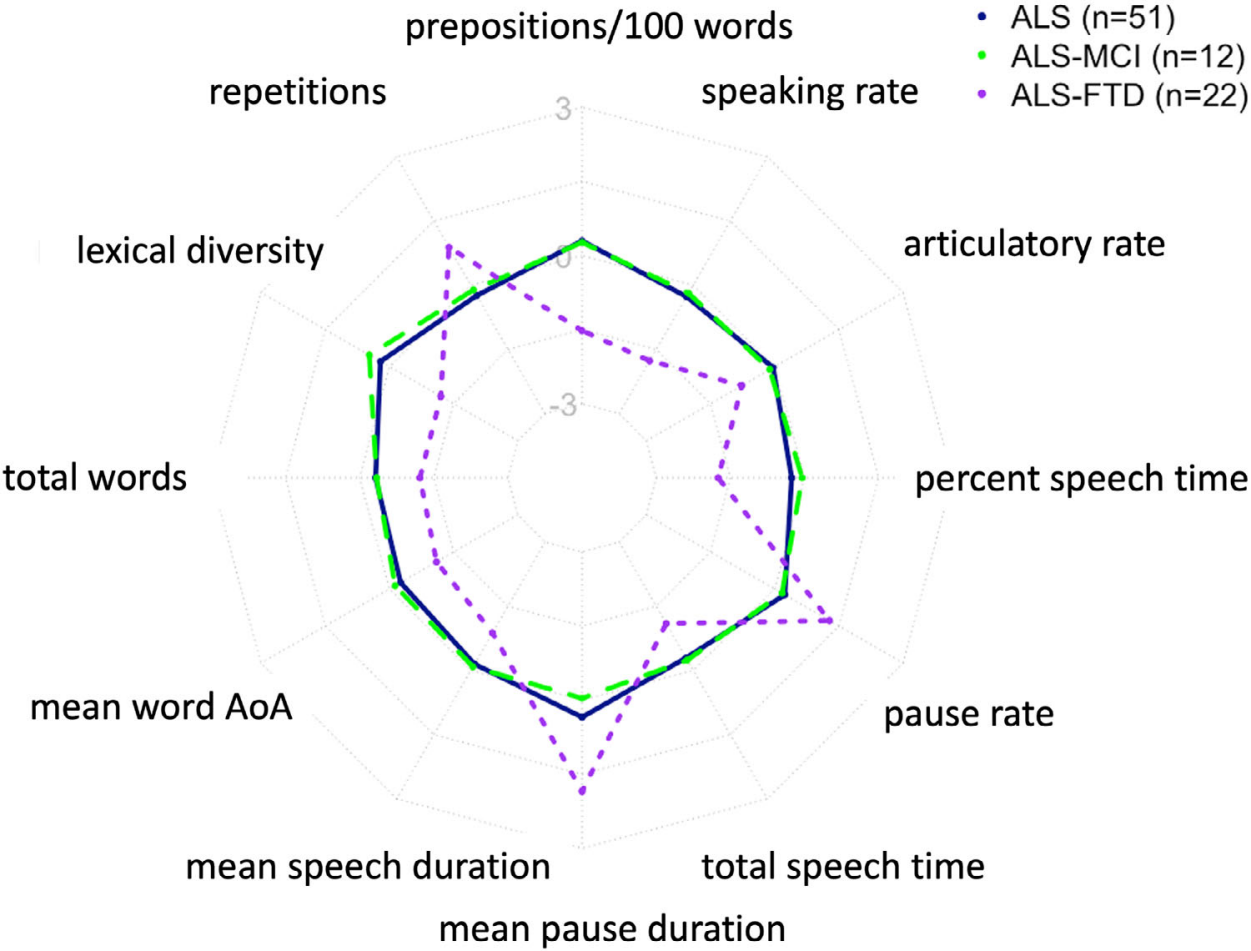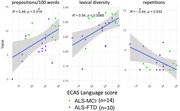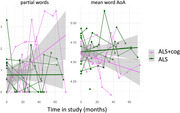# Digital speech markers of cognitive impairment in ALS‐FTD spectrum disorders

**DOI:** 10.1002/alz.089943

**Published:** 2025-01-09

**Authors:** Sanjana Shellikeri, Sunghye Cho, Sharon Ash, Carmen Gonzalez‐Recober, Katheryn A Q Cousins, Corey T McMillan, Lauren Elman, Colin Quinn, Defne A Amado, Michael Baer, David J Irwin, Lauren Massimo, Mark Y Liberman, Naomi Nevler

**Affiliations:** ^1^ Penn FTD Center, University of Pennsylvania, Philadelphia, PA USA; ^2^ Linguistic Data Consortium, University of Pennsylvania, Philadelphia, PA USA; ^3^ Hospital of the University of Pennsylvania, Philadelphia, PA USA; ^4^ University of Pennsylvania, Philadelphia, PA USA; ^5^ University of Pennsylvania, School of Nursing, Philadelphia, PA USA

## Abstract

**Background:**

Comorbid cognitive impairment in amyotrophic lateral sclerosis (i.e., ALS‐FTD), is associated with adverse clinical outcomes and survival. It often exhibits clinical features of behavioral variant frontotemporal dementia (bvFTD) and/or non‐fluent agrammatic primary progressive aphasia (naPPA). Traditional cognitive assessments, such as the Edinburgh Cognitive Assessment Scale (ECAS), require trained personnel for administration. An additional repeatable, objective approach involves automatic analysis of picture descriptions. Prior research identified acoustic and lexical‐semantic impairments in bvFTD, PPAs, and ALS‐FTD (acoustics). This study extends the analysis to the complete speech feature set (n=30) in ALS‐FTD spectrum disorders.

**Method:**

ANCOVAs (FDR adjusted) compared picture descriptions in n=51 ALS, n=22 ALS‐FTD (n=10 bvFTD‐dominant, n=4 naPPA‐dominant), and n=12 ALS with ECAS‐defined mild cognitive/behavioural impairment (ALS‐MCI). Backward selection identified the best feature subset. Post‐hoc assessments examined associations with ECAS behavioral, language, and executive scores. Longitudinal analysis compared within‐individual change over time in n=11 ALS vs. n=9 ALS+cog (combining ALS‐MCI with ALS‐FTD) with available longitudinal data. All models adjusted for bulbar motor scores.

**Result:**

ALS‐FTD significantly differed from ALS and ALS‐MCI on twelve features at first visit: slow speaking and articulatory rates, reduced word count, less percent speech time, shorter speech segments, and longer and more frequent pausing. ALS‐FTD also exhibited fewer adjectives and prepositions, more repetitions, lower lexical diversity, and words with a lower mean age of acquisition (AoA). The best model comprising three features (percent speech, prepositions, and lexical diversity) strongly distinguished ALS‐FTD from ALS (AUC=0.90). ALS‐MCI did not differ from ALS on any speech measure. Acoustic measures were all significantly associated with behavioral, language, and executive scores. Fewer prepositions related to worse behavioral and language scores, and more repetitions and lower lexical diversity related to worse language scores. Mean AoA and articulatory rate were not linked to any ECAS sub‐domains. Longitudinally, ALS+cog exhibited increased partial words and reduced word AoA, while ALS showed no significant change.

**Conclusion:**

Automated speech analysis strongly distinguishes ALS‐FTD from ALS, declines with cognitive severity, and captures distinctive longitudinal changes in ALS‐FTD. These results highlight the potential of automated speech analysis as an objective tool for assessing cognitive impairment in ALS.